# A comprehensive dataset of published records of Leptodirini (Coleoptera, Leiodidae) from the North-western Balkans

**DOI:** 10.3897/BDJ.14.e177525

**Published:** 2026-02-03

**Authors:** Teo Delić, Maja Zagmajster

**Affiliations:** 1 SubBioLab, Department of Biology, Biotechnical Faculty, University of Ljubljana, Ljubljana, Slovenia SubBioLab, Department of Biology, Biotechnical Faculty, University of Ljubljana Ljubljana Slovenia

**Keywords:** subterranean beetles, Leptodirini, North-western Balkans, Dinaric Karst, species richness, distribution ranges, maximum linear extent

## Abstract

**Background:**

In Europe, the beetle tribe Leptodirini (Coleoptera, Leiodidae) reaches its highest diversity in southern parts of the continent, where this predominantly subterranean group developed into one of the largest subterranean radiations in the World. Within this belt, encompassing Iberian Peninsula, Italian Preapls and Balkan Peninsula, the highest species diversity is reached in the North-western Balkan’s Dinaric Karst. Ever since the scientific description of the first subterranean beetle species, *Leptodirus hochenwartii* Schmidt, 1832 in the North-western Dinarides, numerous discoveries and publications accumulated up to present. These records have been systematically gathered in the Slovenian SubBioLab’s database SubBioDB; however, they were previously not publicly available. Herein, we present the dataset on Western Balkan’s Leptodirini and provide some of its basic characteristics.

**New information:**

We present the first comprehensive dataset of published records of the tribe Leptodirini in the North-western Balkans, covering primarily the Dinaric Karst and adjacent areas of the Southern Calcareous Alps and the Albanides. The dataset includes 5,535 records compiled from 333 literature sources representing 334 species with 176 subspecies. All records were georeferenced with the highest possible spatial precision. Using published data, we provide checklists of species and subspecies for included countries, a consolidated bibliography of all publications containing Leptodirini records, spatial maps highlighting the areas of highest taxa richness and an overview of the number of records, sites and range sizes for each species and subspecies across the study area. This dataset provides a foundation for future research and sampling campaigns in the region, as well as a spatially-explicit resource for investigating the diversity and distribution of Leptodirini in Europe’s largest karst area.

## Introduction

The coleopteran tribe Leptodirini (Coleoptera, Leiodidae) represents one of the largest known radiations of subterranean organisms in the world (Antunes‐Carvalho et al. 2017). Except for about ten species that retain reduced number of ommatidia, more than 1300 Leptodirini species are characterised by a complete lack of eyes (Fresneda et al. 2024). In addition, these beetles are typically characterised by a lack of body pigmentation and reduced wings. The three morphological traits are presumably strongly associated with subterranean life and are commonly referred to as troglomorphies (Christiansen 1961, Barr 1968, Culver and Pipan 2019).

**Figure 1.**
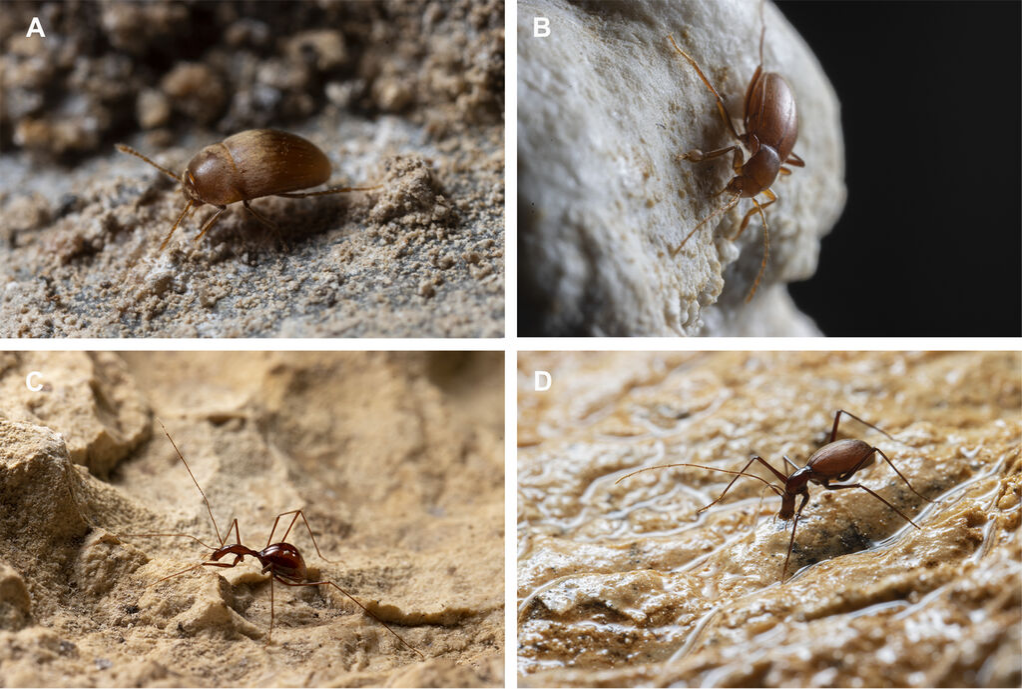
Morphological diversity of the North-western Balkans Leptodirini representatives; **A** *Bathyscimorphus sagarum*; **B** *Oryotus raduhensis*; **C** *Graciliella metohijensis*; **D** *Hadesia vasiceki*.

Leptodirini beetles occupy a wide range of terrestrial environments, from surface habitats to deep subterranean systems. While the vast majority of species are terrestrial, some have also adapted to aquatic habitats (Giachino and Vailati 2006, Giachino and Vailati 2010). A few lineages have independently specialised to the so-called cave hygropetric habitat (Delić et al. 2024). These semi-aquatic environments are characterised by flowing water and rich microbial communities that serve as a primary food source (Sket 2004, Summers Engel et al. 2013). Ecological differences of the tribe representatives are presumably reflected on to their morphologies: species inhabiting surface habitats are typically small and rounded, measuring 2–3 mm, whereas those dwelling in deep caves are considerably larger (8–10 mm) and exhibit distinctive adaptations, such as elongated legs and antennae, as well as modified abdominal structures (Fig. 1).

Comparisons of Leptodirini within Europe revealed the highest species richness and greatest morphological diversification in the Iberian Peninsula, the Italian Prealps and the Dinaric Karst (Fig. 1) (Giachino and Peck 1998, Cieslak et al. 2014, Hlaváč et al. 2017). The first Leptodirini species to be discovered was also the world’s first scientifically described obligate subterranean organism: *Leptodirus hochenwartii* Schmidt, 1832. It was collected in 1831 in Postojnska jama (Slovenia) and formally described a year after (Polak 2005). This landmark discovery in the Dinaric Karst triggered a wave of research in karst landscapes dominated by carbonate rocks and rich in caves, both across Europe and globally. As a result, a new scientific discipline emerged — speleobiology or subterranean biology, the study of subterranean life. Beetles received most attention and already at the start of 20^th^ century, monographs on Leptodirini were published by coleopterologists Jeannel and Müller (Polak 2005). Thanks to a long tradition of speleobiological research in the karst of the North-western Balkans, particularly in the Dinaric Karst, information on Leptodirini started to accumulate in numerous published sources. These range from local, sometimes obscure journals in local languages, to articles in internationally recognised journals. Since the 1980s, records of subterranean fauna, including beetles from this region, started to be systematically collected, which enabled first regional comparisons (Sket et al. 2004). About two decades ago, the spreadsheets were reorganised in a relational spatial database SubBioDB, maintained by the SubBioLab at the Department of Biology, Biotechnical Faculty, University of Ljubljana. This enabled the first spatial analyses of richness patterns in the region, which were conducted on troglobiotic beetles, including Leptodirini (Zagmajster et al. 2008, Zagmajster et al. 2010). The updated dataset was used further, in analyses of factors that shaped the troglobiotic beetle biodiversity patterns (e.g. Bregović and Zagmajster (2016), Bregović et al. (2019)). As new works on Leptodirini continue to be published, including the discovery of new species and subspecies (Čeplík 2023, Delić et al. 2024, Perreau and Hlaváč 2024, Ćurčić et al. 2025), the dataset is constantly being updated and refined. At the same time, these developments highlight the need for a comprehensive regional overview. We therefore believe that the assembly and publication of all available records on Leptodirini from this region represent a much-needed step to support future research and strengthen conservation efforts.

## Project description

### Title

A comprehensive dataset of published records of Leptodirini (Coleoptera, Leiodidae) from the North-western Balkans

### Personnel

Teo Delić, Maja Zagmajster

### Study area description

**Figure 2.**
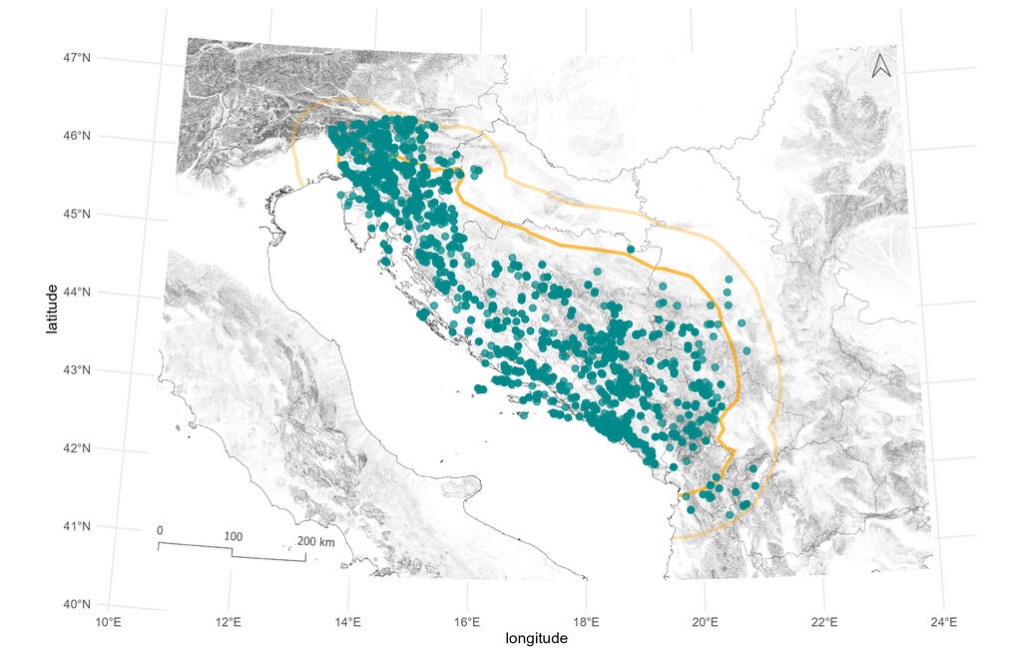
Geographical extent of the presented study, encompassing North-western Balkans with the outline of the Dinaric Karst shown in orange and a 60 km-wide buffer area, used in the analyses, shown in light orange. Leptodirini localities are presented as green dots.

Given the exceptionally high species richness and morphological diversity of Leptodirini in the subterranean environments of the North-western Balkans, our study area is primarily focused on the Dinaric Karst. Extending roughly 650 km in length, Dinaric Karst represents the largest continuous carbonate ridge in Europe. The area was largely shaped by orogenic processes during the Miocene (23.03–5.33 MYA) and, due to the water-soluble nature of carbonate rocks, is characterised by a wide array of karstic features, most notably caves. More than 25,000 caves are estimated to exist within this region. Due to the Dinaric Karst’s close involvement in the orogenic evolution of the Alpine–Dinaric–Carpathian arc (Ustaszewski et al. 2008), which strongly influenced regional biogeography, our study area also includes a 60 km-wide buffer zone encompassing parts of the Southern Limestone Alps in the north and the Albanides in the south (Fig. 2).

## Geographic coverage

### Description

The dataset covers the area of the North-western Balkans, including the area of the Dinaric Karst and limited parts of the neighbouring areas, presented by south-eastern parts of the Southern Limestone Alps and the Northern Albanides.

### Coordinates

41.43 and 46.50 Latitude; 13.27 and 20.91 Longitude.

## Taxonomic coverage

### Description

Presented dataset comprises data on 77 genera, 334 species and 176 subspecies of Leptodirini Lacordaire, 1854. All except one, namely *Phaneropella lesinae* (Reitter, 1881), which is known from both sides of Adriatic Sea (Casale et al. 1991), are endemic to the study region.

### Taxa included

**Table:** 

Rank	Scientific Name	Common Name
kingdom	Animalia	Animals
subkingdom	Eumetazoa	Eumetazoans
phylum	Arthropoda	Arthropods
class	Insecta	Insects
order	Coleoptera	Beetles
superfamily	Staphylinoidea	
family	Leiodidae	Round fungus beetles
subfamily	Cholevinae	
tribe	Leptodirini	
genus	*Adelopidius* Apfelbeck, 1907	
genus	*Adelopsella* Jeannel, 1908	
genus	*Albanella* Müller, 1914	
genus	*Albaniola* Jeannel, 1924	
genus	*Albanodirus* Giachino & Vailati, 1998	
genus	*Anillocharis* Reitter, 1903	
genus	*Anisoscapha* Müller, 1917	
genus	*Anthroherpon* Reitter, 1889	
genus	*Antrodulus* Knirsch, 1927	
genus	*Antrosedes* Reitter, 1912	
genus	*Aphaobiella* Pretner, 1949	
genus	*Aphaobius* Abeille de Perrin, 1878	
genus	*Aphaotus* Breit, 1914	
genus	*Apholeuonus* Reitter, 1889	
genus	*Astagobius* Reitter, 1886	
genus	*Augustia* Zariquiey, 1927	
genus	*Bathyscia* Schiødte, 1848	
genus	*Bathyscidius* Jeannel, 1910	
genus	*Bathyscimorphus* Jeannel, 1910	
genus	*Bathysciopsis* Müller, 1941	
genus	*Bathysciotes* Jeannel, 1910	
genus	*Blattochaeta* Reitter, 1910	
genus	*Ceuthmonocharis* Jeannel, 1910	
genus	*Ceuthophyes* Jeannel, 1924	
genus	*Charonites* Apfelbeck, 1907	
genus	*Croatodirus* Casale, Giachino & Jalžić, 2000	
genus	*Dalmatiola* Jeannel, 1924	
genus	*Deelemaniella* Perreau, 2002	
genus	*Graciliella* Njunjić et al., 2016	
genus	*Hadesia* Müller, 1911	
genus	*Haplotropidius* Müller, 1903	
genus	*Hoffmannella* Müller, 1912	
genus	*Hygrodromus* Giachino, Casale & Jalžić, 2021	
genus	*Icharonia* Reitter, 1912	
genus	*Katobatizon* Knirsch, 1928	
genus	*Kircheria* Giachino & Vailati, 2006	
genus	*Laneyriella* Gúeorguiev, 1976	
genus	*Leonhardella* Reitter, 1903	
genus	*Leonhardia* Reitter, 1901	
genus	*Leptodirus* Schmidt, 1832	
genus	*Leptomeson* Jeannel, 1924	
genus	*Lotharia* Mandl, 1944	
genus	*Magdelainella* Jeannel, 1924	
genus	*Muelleriola* Giachino & Vailati 2019	
genus	*Nauticiella* Moravec & Mlejnek, 2002	
genus	*Oryotus* Miller, 1856	
genus	*Parapropus* Ganglbauer, 1899	
genus	*Pavicevicia* Perreau, 2008	
genus	*Perreauia* Fresneda et al., 2024	
genus	*Phaneropella* Jeannel, 1910	
genus	*Pholeuodromus* Breit, 1913	
genus	*Pholeuonella* Jeannel, 1910	
genus	*Pholeuonopsis* Apfelbeck, 1901	
genus	*Pretneria* Müller, 1931	
genus	*Prokletijella* Perreau & Hlaváč, 2024	
genus	*Proleonhardella* Jeannel, 1910	
genus	*Prospelaeobates* Giachino & Etonti, 1996	
genus	*Protobracharthron* Reitter, 1899	
genus	*Radziella* Casale & Jalžić, 1988	
genus	*Redensekia* Karaman, 1953	
genus	*Remyella* Jeannel, 1931	
genus	*Riberius* Giachino & Casale, 2022	
genus	*Roubaliella* Jeannel, 1925	
genus	*Rozajella* Ćurćić, Brajković & Ćurćić, 2007	
genus	*Rudogorites* Polak & Mulaomerović, 2021	
genus	*Serbiana* Fresneda et al., 2024	
genus	*Setnikia* Breit, 1913	
genus	*Spelaeobates* Müller, 1901	
genus	*Spelaeodromus* Reitter, 1884	
genus	*Spelaites* Apfelbeck, 1907	
genus	*Speonesiotes* Jeannel, 1910	
genus	*Speoplanes* Müller, 1911	
genus	*Sphaerobathyscia* Müller, 1917	
genus	*Tartariella* Nonveiller & Pavićević, 1999	
genus	*Velebitodromus* Casale, Giachino & Jalžić, 2004	
genus	*Weiratheria* Zariquiey, 1927	
genus	*Zariquieyella* Jeannel, 1929	

## Temporal coverage

### Notes

From 1832 to 2025

**Figure 3.**
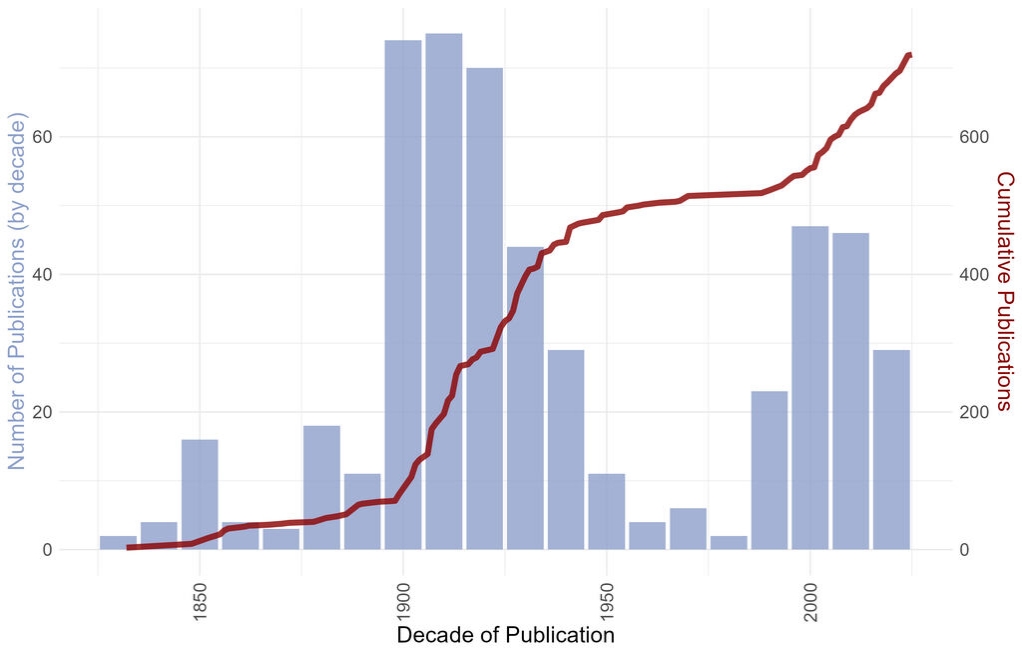
Combined plot showing a histogram of number of publications by the respective decade presented in blue and a dark red line showing a cumulative number of publications through years.

The dynamics of publications, as shown by the pace of publication (Fig. 3), was not even throughout the history of subterranean biology. The oldest record is based on the historical description of *Leptodirus hochenwartii* by Schmidt (1832). Two peaks of publication activity can be distinguished, one at the beginning of the 20^th^ century, from 1900 to 1930 and the second one starting at 1990 and lasting until the present time. The first peak roughly coincides with the general boost in explorations of subterranean organisms. At the time, most of the North-western Balkan countries were a part of the historical, Austro-Hungarian Empire, where natural sciences were highly developed. The mid-century low point might reflect the after-war society crisis and the following political isolation of North-western Balkan countries, when publishing the results in journals was not a prerequisite for prosperous academic career. Finally, the second peak was supposedly triggered by the disintegration of Yugoslavia and opening of the local researchers to international communities, including demand for scientific publications. Additionally, foreign research expeditions to the area started to be more common.

## Usage licence

### Usage licence

Open Data Commons Attribution License

## Data resources

### Data package title

Published records of North-western Balkans Leptodirini

### Resource link

https://doi.org/10.15468/kdzsts

### Number of data sets

1

### Data set 1.

#### Data set name

Published_records_of_Northwestern_Balkans_Leptodirini

#### Data format

TSV (tab-separated) text file

#### Character set

UTF-8

#### Download URL

https://www.gbif.org/dataset/6286d149-28b8-43eb-ae1c-b4bc4ca4789b; https://ipt.pensoft.net/resource?r=published_records_of_northwestern_balkans_leptodirini

#### Data format version

Darwin Core archive

#### Description

The dataset contains 5,535 occurrence records encompassing 334 species and 176 subspecies of Leptodirini, spanning over 1,800 distinct localities (Delić and Zagmajster 2025). Comprehensive details are provided in the “Description of the dataset” section of the Additional Information.

**Data set 1.** 

Column label	Column description
occurrenceID	An occurrence identifier constructed from a combination of the host institution name and a serial number.
InstitutionCode	An acronym and a name for the source institution.
collectionCode	An acronym and a name for the source collection.
informationWithheld	Additional information might exist, but has not been shared in the record (e.g. sensitive or restricted data).
datasetName	The name of the dataset from which the occurrence record derives.
basisOfRecord	The specific nature of the record.
dcterms:bibliographicCitation	A URL (or identifier) pointing to an external resource with more information about the record.
taxonID	An identifier for the taxon (taxonomic unit), constructed from a combination of a host institution name and a serial number.
phylum	The full scientific name of the phylum or division in which the species is classified.
class	The full scientific name of the class in which the taxon is classified.
order	The full scientific name of the order in which the taxon is classified.
family	The full scientific name of the family in which the taxon is classified.
subfamily	The full scientific name of the subfamily in which the taxon is classified.
tribe	The full scientific name of the tribe in which the taxon is classified.
genus	The name of the genus in which the taxon is classified.
specificEpithet	The name of the first or species epithet of the scientificName.
infraspecificEpithet	The name of the second or subspecies epithet of the scientificName.
scientificName	The full scientific name, with authorship and date information.
scientificNameAuthorship	Scientific authorship and date information.
taxonRank	The taxonomic rank of the specific name in the scientificName.
taxonRemarks	Notes on the taxon, for example, whether taxon is considered to be a troglobiont (tgb), a non-troglobiont (ntgb) or undefined (tgb/ntgb).
LocationID	A locality identifier constructed from a combination of the host institution name and a serial number.
Locality	Normalised location string for the location, usually as stated in the source.
decimalLatitude	The geographic latitude (in decimal degrees) of a location.
decimalLongitude	The geographic longitude (in decimal degrees) of a location.
habitat	A category of the habitat where the occurrence was recorded.
country	Name of the country in which the location occurs.
coordinateUncertaintyInMetres	Maximum radius of uncertainty (in metres) around a reported geographic coordinate.
geodeticDatum	The geodetic datum or spatial reference system used by decimalLatitude/decimalLongitude values; WGS-84.
georeferencedBy	The person, group or organisation responsible for determining the georeference (coordinates).
georeferenceProtocol	The methods or protocol used to determine the spatial location and associated uncertainty.

## Additional information

### Sources of occurrences and georeferencing

We extracted the records on the North-western Balkan’s subterranean Leptodirini from published works and organised them in the SubBioDB database, a data management tool of the SubBioLab at the Department of Biology, Biotechnical Faculty, University of Ljubljana. Each record consists of a unique combination of taxon, its locality and the source of information (publication). Each locality was spatially positioned to the greatest possible accuracy, depending on the quality of the description in the source. We georeferenced localities assigning them geographic coordinates according to the following approaches: i) using high precision coordinates published in the articles or determining exact position of the site (cave entrance) using public registries or maps or ii) georeferencing localities according to the closest recognised toponym used in the published work. In the latter case, we coded the classes of coordinates uncertainty by expressing the radius of potential maximal error for each locality; 10 m, 100 m, 1 km, 10 km and 50 km. Only localities with an accuracy up to 10 km were included in the analyses of species richness patterns, localities richness patterns, publication records richness patterns and range size calculations.

### Taxonomy and nomenclature

As the taxonomy of the group developed through the years, raising its complexity with the novel discoveries, curation of taxonomical status was inevitable. We corrected the taxonomy according to the most recent taxonomic status of taxa (Perreau 2000, Hlaváč et al. 2017, Fresneda et al. 2024).

### Description of the dataset

The dataset comprises published records of the predominantly subterranean beetle tribe Leptodirini found in the North-western Balkans, mainly the Dinaric Karst and its vicinity. It contains 5,535 records, related to over 1800 localities; of the latter, 5380 records, referring to 1682 localities, were geographically positioned with an accuracy of up to 10 km. The dataset covers 334 species with 176 subspecies of Leptodirini, extracted from 333 published references. Only 12 of these taxa (8 species with 4 subspecies) were defined as non-troglobiotic, not specialised for life in subterranean environments, whereas 498 (326 species with 172 subspecies) were recognised as taxa bound to live exclusively in the subterranean realm, the so-called troglobionts (Giachino and Vailati 2017).

**Figure 4.**
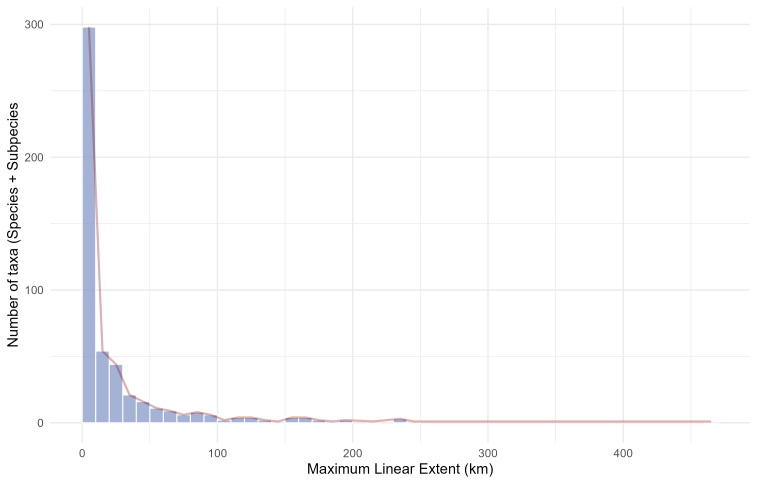
Frequency histogram of taxa distributions according to the maximum linear extent expressed in kilometres (km). Bars show the number of species in 10 km distance bins, while the pale red line represents the summarised number of species at each bin’s mid-point.

Most of the Leptodirini taxa have narrow distribution ranges, 176 (35%) being known from a single site – single site endemics (Fig. 4). This is a bit more than 31% of single site endemics, reported in Bregović et al. (2019), who calculated this for subterranean beetles of two families. Leptodirini distribution ranges, expressed as the maximum linear extent between most distant localities, were between 0 to 464 km, with the 25^th^ percentile at 0.0 km, the median at 5.45 km and the 75^th^ percentile at 25.16 km (Fig. 4). *Bathyscia montana*, a non-troglobiotic beetle, occurring in Bosnia and Herzegovina, Croatia and Slovernia, has the largest distributional range (464 km), while the troglobiotic beetle with the largest distributional range (275 km) is *Leptodirus hochenwartii*, occurring in Slovenia, Croatia and Italy.

**Figure 5.**
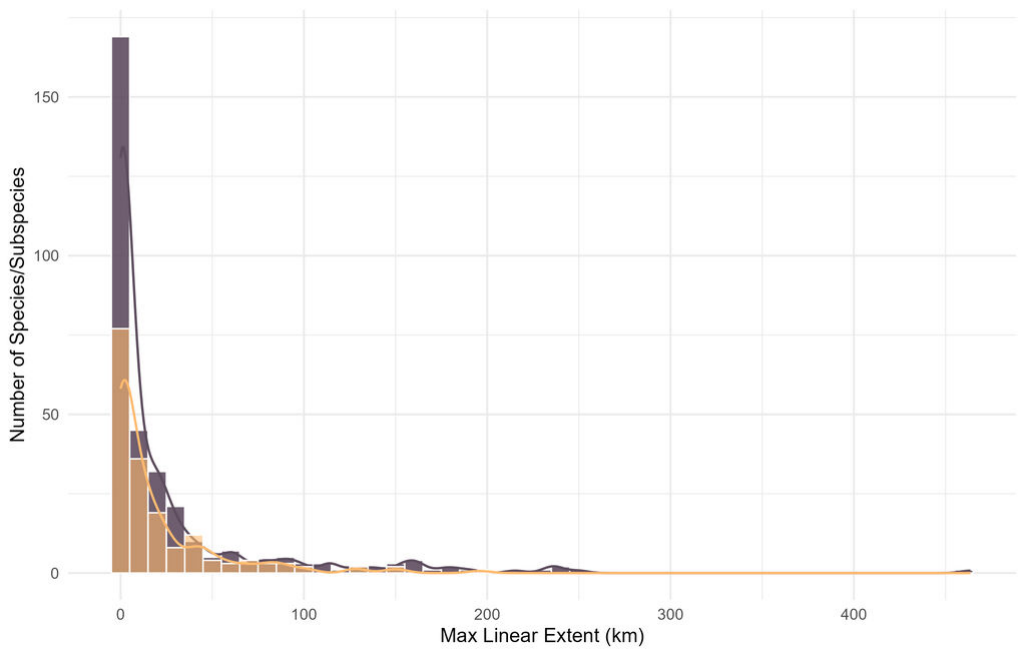
Histogram of species and subspecies distributions by a maximum linear extent expressed in kilometres (km). Bars show the frequency of species (purple) and subspecies (golden) across 10 km distance bins, while the light purple and light gold line represents the summarised number of species and subspecies at each bin’s mid-point, respectively.

**Figure 6.**
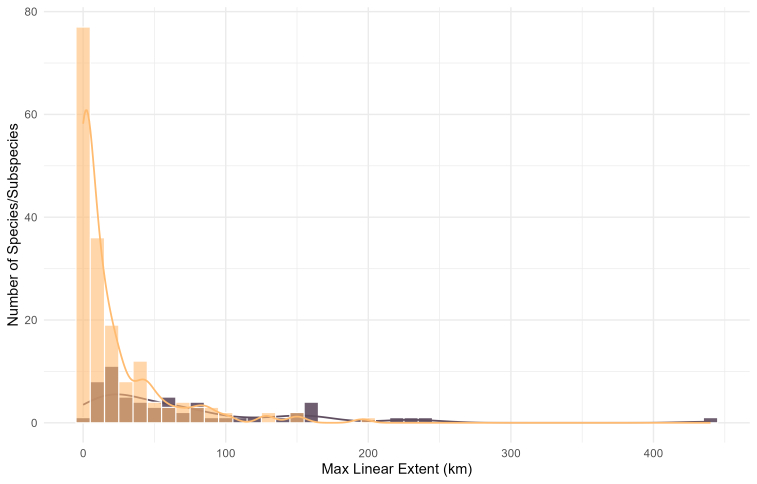
Histogram of maximum linear extent for species (n = 58) which have described subspecies (n = 176). Bars show the frequency of species and subspecies across 10 km distance bins, purple and light gold, while the light purple and light gold lines represent the summarised number of species and subspecies at each bin’s mid-point, respectively.

Similar patterns in range size can be seen when species and subspecies are presented separately (Fig. 5). At specific level, the 25^th^ percentile remains at 0.0 km, the median value drops to 4.7 km and the 75^th^ percentile to 25.2 km (Fig. 5). At the subspecies level, distributional ranges become even smaller. The largest range, 196 km, is met at the subspecies *Bathyscia montana montana*; the 25^th^ percentile is 0.0 km, the median grows to 7.8 km and the 75^th^ percentile to 24.8 km (Fig. 5). Finally, if we compare the sizes of distributional ranges only in species with the existing subspecies (58 species with 176 subspecies), the species range size pattern is different. Species with many subspecies described generally have larger ranges and there are only two out of 58 species that retain range size smaller than 10 km (Fig. 6). On the other hand, most of the subspecies have range sizes in this size class (0 – 10 km).

**Figure 7.**
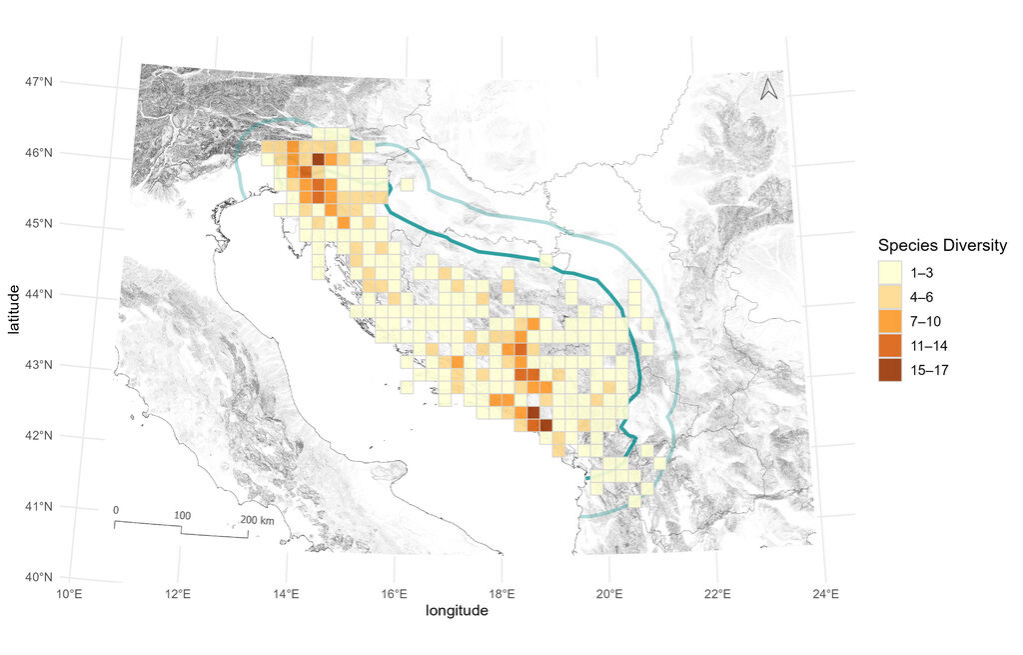
Patterns of species richness, based on published data on Leptodirini, shown on a 20 x 20 km cell grid covering the Dinaric Karst (dark green) and the 60 km buffer area (light green).

**Figure 8.**
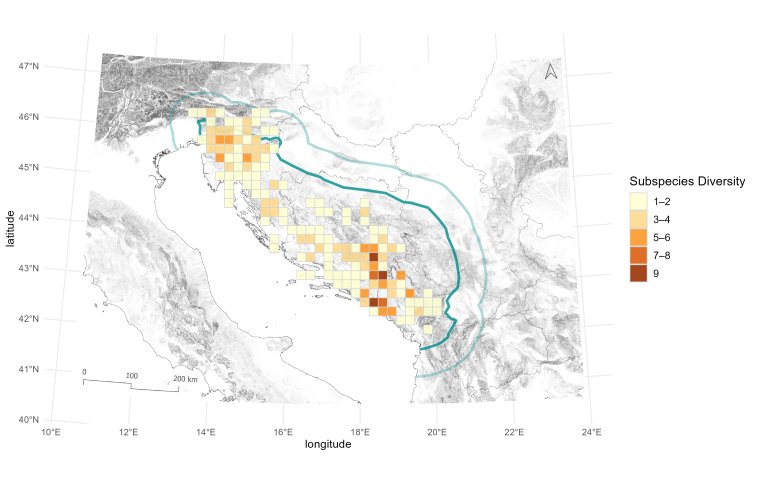
Patterns of subspecies richness, based on published data on Leptodirini, shown on a 20 x 20 km cell grid covering the Dinaric Karst (dark green) and the 60 km buffer area (light green) used in the analysis.

**Figure 9.**
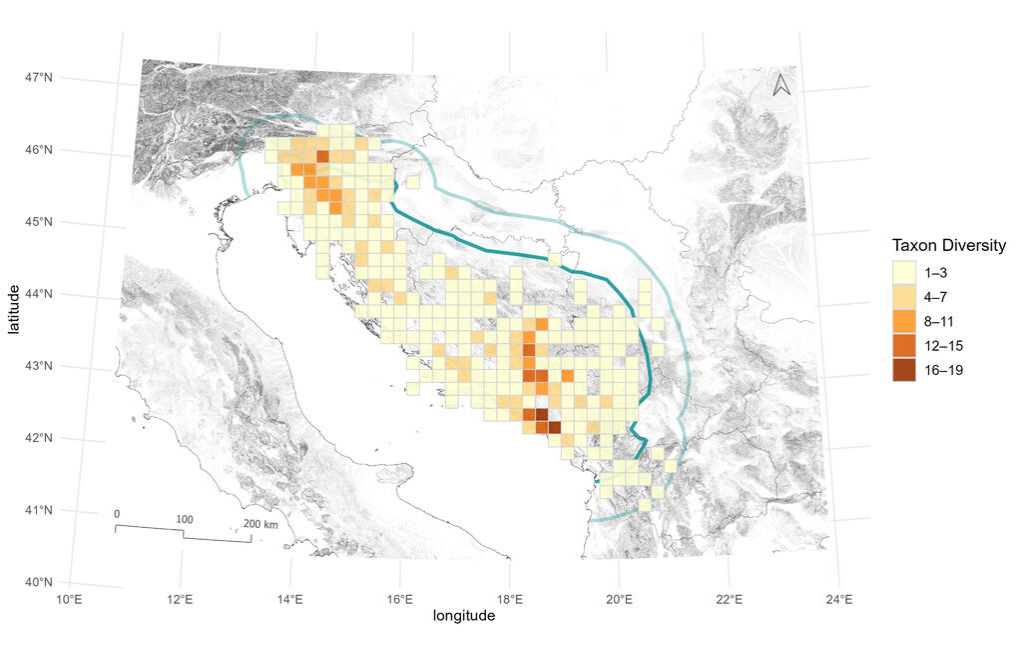
Patterns of species and subspecies richness, based on published data on Leptodirini, shown on a 20 x 20 km cell grid covering the Dinaric Karst (dark green) and the 60 km buffer area (light green) used in the analysis.

Biodiversity richness patterns of Leptodirini are not uniform, as 20 x 20 km cells contain different numbers of Leptodirini. Cells with high species richness occur in two areas, one in north-western and the other in south-eastern part of the region. This pattern was already shown in Dinaric subterranean beetles (Zagmajster et al. 2008, Bregović and Zagmajster 2016); therefore, our study confirms the existence of two separate hotspots also in Dinaric Leptodirini. The cells with the highest number of species, counting 15 and 17 species, can be found in the Northern and Southern Dinarides, respectively (Fig. 7). On the subspecies level, the highest diversity of described species is met in the Southern Dinarides, in south-eastern Herzegovina, where the number of described subspecies reaches up to nine (Fig. 8). When combined, the spatial visualisation of the two datasets reveals a somewhat different pattern (Fig. 9), amongst other issues, also calling into question the taxonomic validity of the currently recognised subspecies and the need to revise their taxonomic status or their synonymisation with existing species.

**Figure 10.**
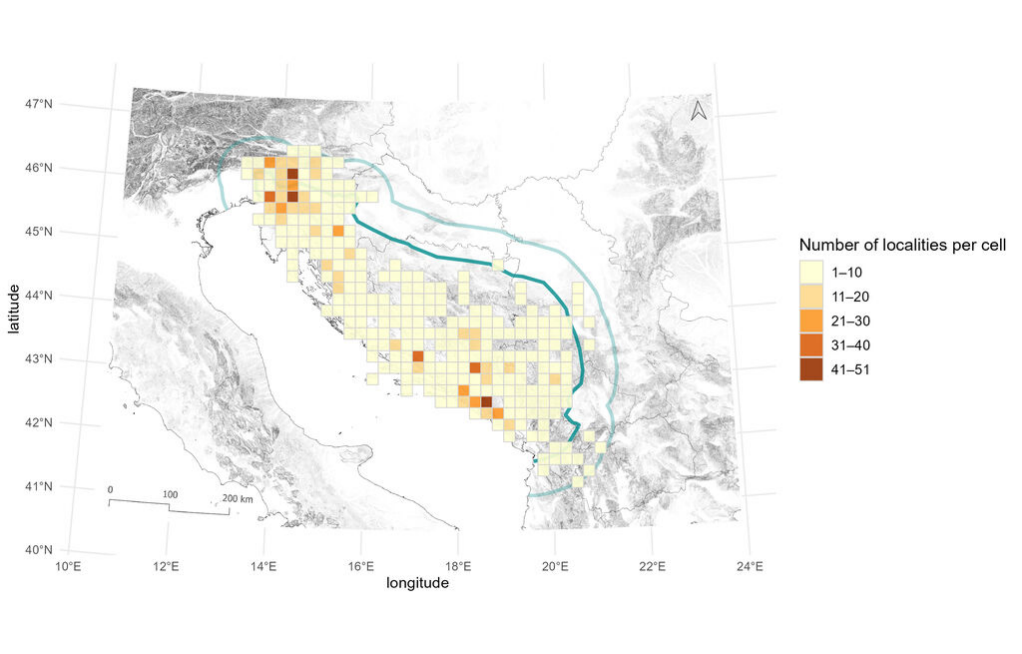
Overview of the number of localities per 20 × 20 km grid cell with recorded occurrences of *Leptodirini* in the North-western Balkans. The study area includes the Dinaric Karst (dark green) and the surrounding 60 km buffer zone (light green) used in the analysis.

**Figure 11.**
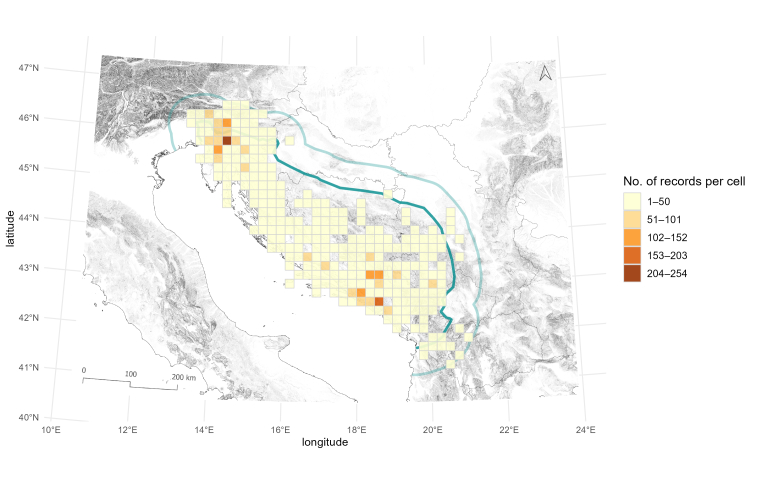
Colour-coded overview of the number of literature records per 20 x 20 km grid cell overlaid on the North-western Balkans. The study area includes the Dinaric Karst (dark green) and the surrounding 60 km buffer zone (light green) used in the analysis.

Similarly, we checked the number of localities per grid cell. The number of localities per cell ranges from one to 51. As in the species richness analyses, the two centres of sampling, in the Northern and Southern Dinarides, can be distinguished (Fig. 10). Fifty percent of grids, with published data, have three or fewer localities sampled, while 75% of all grid cells have eight or fewer localities sampled. Notably, cells with high species or taxa richness and cells with many localities sampled overlap to a certain extent. This confirms that the observed richness pattern cannot be explained by differences in the number of sampling localities, even though they do exist (see Zagmajster et al. (2010)). Finally, we checked for the differences in published records of Leptodirini in the North-western Balkans. Our analysis shows that the published records are dispersed highly unevenly. Fifty percent (n = 139) of grid cells with species have one to eight records, whereas only seven cells have more than 100 records (Fig. 11, Tables 1, 2). The grid cells with the highest number of records correspond to cells where the known and historically most surveyed caves are situated, Postojnska jama and Vjetrenica, counting 254 and 186 records, respectively.

The assembled dataset enabled preparation overviews of genera, species and subspecies currently reported in each of the studied countries. These lists present a checklist of Leptodirini species for five countries of the Dinaric Karst, namely Bosnia and Herzegovina, Croatia, Kosovo, Montenegro and Slovenia. As such, they provide a much-needed baseline to evaluate new findings, either extending the known distribution ranges or adding new species country specific faunistic lists. For the rest of the countries (Albania, Austria, Italy, North Macedonia and Serbia), these numbers are not complete, as Leptodirini fauna in larger parts of these countries can be attributed to other geomorphological units, i.e. Alps, Albanides, Rhodopes etc., which are known to have their own fauna (Fresneda et al. 2024).

## Figures and Tables

**Table 1.** List of species as reported in each of the countries of the Dinaric Karst and the adjacent areas. Please note that only results for the countries in bold can be taken as representative, since only a smaller portion of other countries were included in the analysis. The countries are abbreviated in the following way: ALB – Albania, AUT – Austria, BIH – Bosnia and Herzegovina, CRO – Croatia, ITA – Italy, KOS – Kosovo, MNE – Montenegro, NMK – North Macedonia, SRB – Serbia, SLO – Slovenia.

	ALB	AUT	**BIH**	**CRO**	ITA	KOS	**MNE**	SRB	**SLO**	NMK
**Total number of species reported per country**	19	5	**134**	**70**	11	1	**71**	22	**66**	4
*Adelopidius boksevicensis*			✔							
*Adelopidius bufonis*			✔							
*Adelopidius hrustovacensis*			✔							
*Adelopidius kuchtae*			✔							
*Adelopidius ljubacevoensis*			✔							
*Adelopidius neumanni*			✔							
*Adelopidius ovatus*			✔							
*Adelopidius pougarjensis*			✔							
*Adelopidius sequensi*			✔							
*Adelopidius weiratherianus*			✔							
*Adelopsella bosnica*			✔	✔			✔			
*Albanella lonae*	✔						✔			
*Albanella reissi*							✔			
*Albanella scutariensis*							✔			
*Albaniola merditana*	✔									
*Albaniola moraveci*										✔
*Albaniola rambouseki*										✔
*Albanodirus gobettii*	✔									
*Albanodirus ivanpetrovi*	✔									
*Albanodirus trezzii*	✔									
*Anillocharis hawelkai*			✔							
*Anillocharis ottonis*			✔							
*Anillocharis stenopterus*			✔							
*Anillocharis tenuilimbatus*							✔			
*Anisoscapha klimeschi*			✔	✔						
*Anisoscapha winkleri*				✔						
*Anthroherpon brckoensis*			✔							
*Anthroherpon cecai*							✔			
*Anthroherpon charon*			✔							
*Anthroherpon cylindricollis*			✔							
*Anthroherpon elongatum*			✔							
*Anthroherpon erebus*			✔							
*Anthroherpon ganglbaueri*			✔							
*Anthroherpon garbellii*	✔									
*Anthroherpon gueorguievi*							✔			
*Anthroherpon harbichi*			✔							
*Anthroherpon hoermanni*			✔				✔			
*Anthroherpon hossei*			✔							
*Anthroherpon latipenne*	✔		✔				✔			
*Anthroherpon matulici*			✔	✔			✔			
*Anthroherpon matzenaueri*							✔			
*Anthroherpon piesbergeni*							✔			
*Anthroherpon pozi*			✔							
*Anthroherpon primitivum*			✔				✔			
*Anthroherpon pygmaeum*			✔							
*Anthroherpon scutariensis*	✔									
*Anthroherpon shtarensis*	✔									
*Anthroherpon sinjajevina*							✔			
*Anthroherpon spelaebatoides*			✔							
*Anthroherpon stenocephalum*			✔							
*Anthroherpon subalpinum*			✔							
*Anthroherpon taxi*	✔		✔				✔	✔		
*Anthroherpon udrzali*							✔			
*Anthroherpon weiratheri*			✔							
*Anthroherpon winneguthi*			✔							
*Anthroherpon zariquieyi*							✔			
*Antrodulus aequibasalis*			✔							
*Antrodulus occultus*			✔							
*Antrosedes longicollis*			✔							
*Antrosedes speluncarius*			✔							
*Aphaobiella budnarlipoglavseki*									✔	
*Aphaobiella kofleri*									✔	
*Aphaobiella mlejneki*									✔	
*Aphaobiella tisnicensis*									✔	
*Aphaobius ajdae*									✔	
*Aphaobius alphonsi*									✔	
*Aphaobius angusticollis*									✔	
*Aphaobius brevicornis*		✔								
*Aphaobius forojuliensis*					✔				✔	
*Aphaobius fortesculptus*									✔	
*Aphaobius gorenjskanus*									✔	
*Aphaobius grottoloi*					✔					
*Aphaobius haraldi*		✔								
*Aphaobius heydeni*									✔	
*Aphaobius kahleni*									✔	
*Aphaobius kaplai*									✔	
*Aphaobius knirschi*									✔	
*Aphaobius kofleri*									✔	
*Aphaobius kraussi*		✔							✔	
*Aphaobius lebenbaueri*									✔	
*Aphaobius ljubnicensis*									✔	
*Aphaobius luckae*									✔	
*Aphaobius mateji*									✔	
*Aphaobius milleri*				✔					✔	
*Aphaobius miricae*									✔	
*Aphaobius mixanigi*		✔								
*Aphaobius muellerianus*									✔	
*Aphaobius ninae*									✔	
*Aphaobius robustus*									✔	
*Aphaotus cadamuroi*					✔					
*Apholeuonus absoloni*			✔							
*Apholeuonus insignis*			✔							
*Apholeuonus leonhardi*			✔							
*Apholeuonus longicollis*			✔							
*Apholeuonus nudus*			✔							
*Astagobius angustatus*				✔					✔	
*Astagobius hadzii*				✔						
*Augustia weiratheri*			✔							
*Bathyscia montana*			✔	✔	✔				✔	
*Bathyscidius basarai*	✔									
*Bathyscidius crnagorensis*							✔			
*Bathyscidius fallaciosus*				✔						
*Bathyscidius komajiensis*				✔						
*Bathyscidius mikati*							✔			
*Bathyscidius mljetensis*				✔						
*Bathyscidius orjensis*							✔			
*Bathyscidius tristiculus*				✔						
*Bathyscimorphus acuminatus*									✔	
*Bathyscimorphus adriaticus*									✔	
*Bathyscimorphus byssinus*									✔	
*Bathyscimorphus croaticus*				✔						
*Bathyscimorphus globosus*									✔	
*Bathyscimorphus kladniki*									✔	
*Bathyscimorphus likanensis*				✔						
*Bathyscimorphus posarinii*									✔	
*Bathyscimorphus pretneri*									✔	
*Bathyscimorphus sagarum*									✔	
*Bathyscimorphus serkoi*									✔	
*Bathyscimorphus slavkoi*									✔	
*Bathyscimorphus trifurcatus*									✔	
*Bathyscimorphus uskokensis*				✔					✔	
*Bathysciopsis sternalis*			✔							
*Bathysciotes khevenhuelleri*				✔	✔				✔	
*Blattochaeta brankojalzici*							✔			
*Blattochaeta hawelkai*							✔			
*Blattochaeta marianii*			✔	✔			✔			
*Blattochaeta matchai*							✔			
*Blattochaeta montenegrina*							✔			
*Blattochaeta peterhlavaci*							✔			
*Blattochaeta remyi*							✔			
*Ceuthmonocharis freyeri*									✔	
*Ceuthmonocharis matjasici*									✔	
*Ceuthmonocharis netolitzkyi*									✔	
*Ceuthmonocharis pusillus*									✔	
*Ceuthmonocharis robici*									✔	
*Ceuthophyes bischoffi*	✔									
*Ceuthophyes bukoviki*										✔
*Ceuthophyes enormis*	✔									
*Ceuthophyes lazaropolensis*										✔
*Charonites matzenaueri*			✔							
*Charonites orlovacensis*			✔							
*Charonites scheibeli*			✔							
*Charonites weiratheri*			✔							
*Charonites zoppae*			✔							
*Croatodirus bozicevici*				✔						
*Croatodirus casalei*				✔						
*Croatodirus ozimeci*				✔					✔	
*Dalmatiola curzolensis*				✔						
*Deelemaniella pretneri*			✔							
*Graciliella absoloni*							✔			
*Graciliella apfelbecki*	✔		✔	✔			✔			
*Graciliella kosovaci*				✔						
*Graciliella lahneri*			✔				✔			
*Graciliella metohijensis*			✔				✔			
*Graciliella ozimeci*				✔			✔			
*Hadesia asamo*			✔							
*Hadesia lakotai*			✔							
*Hadesia persephonae*							✔			
*Hadesia ticari*							✔			
*Hadesia vasiceki*			✔	✔						
*Hadesia weiratheri*							✔			
*Hadesia zetae*							✔			
*Haplotropidius cadeki*			✔	✔						
*Haplotropidius mariani*			✔							
*Haplotropidius pubescens*			✔	✔						
*Haplotropidius taxi*				✔						
*Haplotropidius vranensis*			✔							
*Hoffmannella makarensis*				✔						
*Hygrodromus nikolinae*				✔						
*Icharonia leonhardiana*			✔							
*Katobatizon antennarium*			✔							
*Katobatizon apfelbecki*			✔							
*Kircheria beroni*	✔									
*Kircheria dritae*	✔									
*Laneyriella andrijevicensis*							✔			
*Laneyriella ganglbaueri*							✔			
*Laneyriella matchai*			✔				✔			
*Laneyriella milotiana*	✔									
*Laneyriella scutariensis*	✔						✔			
*Laneyriella staudacheri*				✔						
*Laneyriella stussineri*							✔			
*Leonhardella angulicollis*			✔							
*Leonhardella antennaria*							✔			
*Leonhardella jeanneli*			✔							
*Leonhardella montenegrina*							✔			
*Leonhardella roseni*							✔			
*Leonhardella setnikana*			✔							
*Leonhardella setniki*			✔				✔			
*Leonhardia delminiumica*			✔							
*Leonhardia droveniki*			✔							
*Leonhardia hilfi*			✔							
*Leonhardia jajcensis*			✔							
*Leonhardia reitteri*			✔							
*Leonhardia sebesicensis*			✔							
*Leonhardia solaki*			✔							
*Leptodirus hochenwartii*				✔	✔				✔	
*Leptomeson biokovensis*				✔						
*Leptomeson bujasi*				✔						
*Leptomeson dalmatinus*				✔						
*Leptomeson dombrowskii*			✔	✔						
*Leptomeson leonhardi*			✔							
*Leptomeson loreki*			✔							
*Leptomeson radjai*				✔						
*Leptomeson raguzi*			✔							
*Leptomeson soltensis*				✔						
*Leptomeson svircevi*			✔							
*Leptomeson vuicae*				✔						
*Lotharia angulicollis*		✔								
*Magdelainella hussoni*							✔	✔		
*Magdelainella kauti*			✔							
*Magdelainella nonveilleri*								✔		
*Magdelainella serbica*			✔				✔	✔		
*Muelleriola sylvestris*				✔					✔	
*Nauticiella djokici*			✔							
*Nauticiella numerosa*							✔			
*Nauticiella stygivaga*			✔							
*Oryotus ausmeieri*									✔	
*Oryotus gasparoi*					✔				✔	
*Oryotus indentatus*					✔				✔	
*Oryotus micklitzi*									✔	
*Oryotus raduhensis*									✔	
*Oryotus schmidtii*				✔					✔	
*Oryotus trezzii*					✔				✔	
*Parapropus brevicollis*			✔							
*Parapropus ganglbaueri*			✔							
*Parapropus insignis*			✔							
*Parapropus jasminkoi*			✔							
*Parapropus neumanni*			✔							
*Parapropus nonveilleri*			✔							
*Parapropus pfeiferi*			✔							
*Parapropus sericeus*			✔	✔					✔	
*Parapropus vitorogensis*			✔							
*Pavicevicia comottiorum*								✔		
*Pavicevicia pretneri*						✔				
*Perreauia dalmatica*				✔						
*Phaneropella lesinae*			✔	✔						
*Pholeuodromus breiti*			✔							
*Pholeuodromus jeanneli*			✔							
*Pholeuonella bosnicola*			✔							
*Pholeuonella erberii*			✔	✔			✔			
*Pholeuonopsis cvijici*								✔		
*Pholeuonopsis ganglbaueri*			✔							
*Pholeuonopsis grabowskii*			✔							
*Pholeuonopsis herculeanus*			✔				✔			
*Pholeuonopsis intermedius*			✔							
*Pholeuonopsis leonhardi*			✔				✔			
*Pholeuonopsis lupi*								✔		
*Pholeuonopsis magdelainei*								✔		
*Pholeuonopsis perucensis*			✔							
*Pholeuonopsis pfeiferi*			✔							
*Pholeuonopsis romanjensis*			✔							
*Pholeuonopsis setipennis*			✔							
*Pholeuonopsis sljivovicensis*								✔		
*Pholeuonopsis spaethi*			✔							
*Pholeuonopsis tarensis*								✔		
*Pholeuonopsis weiratheri*			✔							
*Pholeuonopsis zlatiborensis*								✔		
*Pretneria latitarsis*									✔	
*Pretneria melissae*									✔	
*Pretneria metkae*									✔	
*Pretneria saulii*					✔				✔	
*Pretneria soriscensis*									✔	
*Pretneria ternovensis*									✔	
*Prokletijella montana*							✔			
*Proleonhardella adolfi*			✔							
*Proleonhardella apfelbecki*			✔							
*Proleonhardella epimikes*			✔							
*Proleonhardella hirtella*							✔	✔		
*Proleonhardella leonhardi*			✔							
*Proleonhardella matzenaueri*			✔							
*Proleonhardella neumanni*			✔							
*Proleonhardella remyi*							✔	✔		
*Proleonhardella serbooccidentalis*								✔		
*Proleonhardella tarensis*								✔		
*Proleonhardella weiratheri*			✔							
*Prospelaeobates bognoloi*				✔						
*Prospelaeobates brelihi*				✔					✔	
*Prospelaeobates vrezeci*									✔	
*Protobracharthron dusinae*			✔							
*Protobracharthron reitteri*			✔							
*Radziella styx*				✔						
*Redensekia likana*				✔						
*Remyella hussoni*								✔		
*Remyella javorensis*								✔		
*Remyella propiformis*							✔	✔		
*Remyella raskae*								✔		
*Remyella scaphoides*							✔	✔		
*Remyella spanovicae*								✔		
*Riberius stillicidii*	✔									
*Roubaliella biokovensis*				✔						
*Rozajella deelemani*							✔			
*Rozajella jovanvladimiri*							✔			
*Rozajella madzgalji*							✔			
*Rozajella ognjenovici*							✔			
*Rudogorites simonei*			✔							
*Serbiana latitarsis*								✔		
*Setnikia leonhardi*			✔							
*Spelaeobates bachofeni*				✔						
*Spelaeobates coriniensis*				✔						
*Spelaeobates czernyi*				✔						
*Spelaeobates kraussi*				✔						
*Spelaeobates novaki*				✔						
*Spelaeobates peneckei*				✔						
*Spelaeobates pharensis*				✔						
*Spelaeodromus pluto*				✔						
*Spelaeodromus sneznikensis*									✔	
*Spelaites grabowskii*				✔						
*Speonesiotes brachycerus*							✔			
*Speonesiotes dorotkanus*			✔	✔			✔			
*Speonesiotes gobanzi*				✔						
*Speonesiotes huemmleri*							✔			
*Speonesiotes insularis*				✔						
*Speonesiotes issensis*				✔						
*Speonesiotes koritoensis*			✔							
*Speonesiotes laticollis*							✔			
*Speonesiotes matchai*							✔			
*Speonesiotes montenegrinus*							✔			
*Speonesiotes muelleri*			✔							
*Speonesiotes narentinus*			✔	✔			✔			
*Speonesiotes paganettii*				✔			✔			
*Speonesiotes pretneri*							✔			
*Speonesiotes rambouseki*			✔							
*Speonesiotes remyi*			✔							
*Speonesiotes schweitzeri*			✔							
*Speonesiotes septentrionalis*			✔							
*Speonesiotes spalacis*			✔							
*Speoplanes biocovensis*			✔	✔						
*Speoplanes giganteus*				✔						
*Sphaerobathyscia hoffmanni*					✔				✔	
*Tartariella durmitorensis*							✔			
*Velebitodromus ozrenlukici*				✔						
*Velebitodromus smidai*				✔						
*Weiratheria bocki*							✔			
*Zariquieyella biokovensis*				✔						

**Table 2.** List of subspecies as reported in each of the countries of the Dinaric Karst and the adjacent areas. Please note that only results for the countries in bold can be taken as representative, since only a smaller portion of other countries were included in the analysis. The countries are abbreviated in the following way: ALB – Albania, AUT – Austria, BIH – Bosnia and Herzegovina, CRO – Croatia, ITA – Italy, KOS – Kosovo, MNE – Montenegro, NMK – North Macedonia, SRB – Serbia, SLO – Slovenia.

	ALB	**BIH**	**CRO**	ITA	**MNE**	SRB	**SLO**
**Total number of subspecies reported per country**	6	**95**	**40**	5	**30**	1	**25**
*Albanella lonae lonae*	✔						
*Albanella lonae zoufali*	✔				✔		
*Anillocharis stenopterus matzenaueri*		✔					
*Anillocharis stenopterus stenopterus*		✔					
*Anisoscapha klimeschi klimeschi*		✔	✔				
*Anisoscapha klimeschi misella*			✔				
*Anthroherpon cylindricollis cylindricollis*		✔					
*Anthroherpon cylindricollis scaphium*		✔					
*Anthroherpon cylindricollis thoracicum*		✔					
*Anthroherpon erebus erebus*		✔					
*Anthroherpon erebus scheibeli*		✔					
*Anthroherpon ganglbaueri alticola*		✔					
*Anthroherpon ganglbaueri distinguendum*		✔					
*Anthroherpon ganglbaueri ganglbaueri*		✔					
*Anthroherpon ganglbaueri intermedium*		✔					
*Anthroherpon hoermanni hoermanni*		✔					
*Anthroherpon hoermanni hoffmanni*		✔					
*Anthroherpon hoermanni hypsophilum*		✔					
*Anthroherpon hoermanni orlovacensis*					✔		
*Anthroherpon hoermanni sericeum*		✔					
*Anthroherpon latipenne attenuatum*					✔		
*Anthroherpon latipenne gottli*					✔		
*Anthroherpon latipenne latellai*	✔						
*Anthroherpon latipenne latipenne*		✔			✔		
*Anthroherpon latipenne punctipennis*					✔		
*Anthroherpon matzenaueri augustae*					✔		
*Anthroherpon matzenaueri matzenaueri*					✔		
*Anthroherpon matzenaueri taliensis*					✔		
*Anthroherpon primitivum jeanneli*		✔					
*Anthroherpon primitivum primitivum*		✔					
*Anthroherpon pygmaeum pygmaeum*		✔					
*Anthroherpon pygmaeum stricticolle*		✔					
*Anthroherpon stenocephalum noesskei*		✔					
*Anthroherpon stenocephalum stenocephalum*		✔					
*Anthroherpon taxi albanicum*	✔				✔		
*Anthroherpon taxi boschi*					✔	✔	
*Anthroherpon taxi hercegovinum*		✔					
*Anthroherpon taxi lemur*		✔			✔		
*Anthroherpon taxi muelleri*		✔			✔		
*Anthroherpon taxi pretneri*					✔		
*Anthroherpon taxi remyi*					✔		
*Anthroherpon taxi sydowi*					✔		
*Anthroherpon taxi taxi*		✔			✔		
*Anthroherpon taxi trezzii*	✔						
*Anthroherpon taxi winkleri*					✔		
*Aphaobiella budnarlipoglavseki budnarlipoglavseki*							✔
*Aphaobiella budnarlipoglavseki mozirjensis*							✔
*Apholeuonus longicollis longicollis*		✔					
*Apholeuonus longicollis pretneri*		✔					
*Apholeuonus longicollis sequensi*		✔					
*Apholeuonus nudus cryophilus*		✔					
*Apholeuonus nudus ledenjacensis*		✔					
*Apholeuonus nudus nudus*		✔					
*Apholeuonus nudus petrovici*		✔					
*Apholeuonus nudus sturanyi*		✔					
*Apholeuonus nudus winkleri*		✔					
*Astagobius angustatus angustatus*							✔
*Astagobius angustatus deelemani*			✔				
*Astagobius angustatus driolii*			✔				
*Astagobius angustatus glacialis*							✔
*Astagobius angustatus langhofferi*			✔				
*Astagobius angustatus laticollis*							✔
*Astagobius angustatus vukusici*			✔				
*Bathyscia montana apfelbecki*		✔					
*Bathyscia montana forticornis*							✔
*Bathyscia montana jablanicensis*		✔					
*Bathyscia montana montana*			✔	✔			✔
*Bathyscimorphus acuminatus acuminatus*							✔
*Bathyscimorphus acuminatus ruzickai*							✔
*Bathyscimorphus byssinus byssinus*							✔
*Bathyscimorphus likanensis stilleri*			✔				
*Bathysciotes khevenhuelleri crepsensis*			✔				
*Bathysciotes khevenhuelleri croaticus*			✔				
*Bathysciotes khevenhuelleri horvathi*			✔				
*Bathysciotes khevenhuelleri khevenhuelleri*							✔
*Bathysciotes khevenhuelleri tergestinus*				✔			✔
*Blattochaeta marianii brevipennis*		✔					
*Blattochaeta marianii kusijanovici*			✔				
*Blattochaeta marianii marianii*		✔			✔		
*Blattochaeta marianii paganettii*		✔			✔		
*Ceuthmonocharis netolitzkyi kodrici*							✔
*Ceuthmonocharis netolitzkyi netolitzkyi*							✔
*Ceuthmonocharis robici robici*							✔
*Ceuthmonocharis robici staudacheri*							✔
*Charonites matzenaueri apfelbecki*		✔					
*Charonites matzenaueri matzenaueri*		✔					
*Charonites weiratheri prosternalis*		✔					
*Charonites weiratheri pygmaeus*		✔					
*Charonites weiratheri weiratheri*		✔					
*Graciliella apfelbecki apfelbecki*		✔	✔				
*Graciliella apfelbecki schwienbacheri*	✔						
*Graciliella apfelbecki sculptifrons*		✔					
*Graciliella apfelbecki scutulatum*		✔					
*Haplotropidius mariani cabuljensis*		✔					
*Haplotropidius mariani cvrstnicensis*		✔					
*Haplotropidius mariani mariani*		✔					
*Haplotropidius pubescens livnensis*		✔					
*Haplotropidius pubescens pubescens*			✔				
*Haplotropidius pubescens svilajensis*			✔				
*Haplotropidius taxi novaki*			✔				
*Haplotropidius taxi subinflatus*			✔				
*Haplotropidius taxi taxi*			✔				
*Haplotropidius vranensis heteromorphus*		✔					
*Haplotropidius vranensis vranensis*		✔					
*Icharonia leonhardiana leonhardiana*		✔					
*Icharonia leonhardiana trescavicensis*		✔					
*Leonhardella antennaria acutangula*					✔		
*Leonhardella antennaria antennaria*					✔		
*Leonhardella antennaria brevis*					✔		
*Leonhardella setnikana kyselyi*		✔					
*Leonhardella setnikana setnikana*		✔					
*Leonhardella setniki bielasicensis*		✔					
*Leonhardella setniki setniki*					✔		
*Leonhardia hilfi hilfi*		✔					
*Leonhardia hilfi robusta*		✔					
*Leonhardia reitteri mersa*		✔					
*Leonhardia reitteri reitteri*		✔					
*Leonhardia reitteri zariquieyi*		✔					
*Leptodirus hochenwartii croaticus*			✔				
*Leptodirus hochenwartii hochenwartii*							✔
*Leptodirus hochenwartii pretneri*			✔				
*Leptodirus hochenwartii reticulatus*			✔	✔			✔
*Leptodirus hochenwartii schmidtii*							✔
*Leptodirus hochenwartii velebiticus*			✔				
*Leptomeson dombrowskii dombrowskii*			✔				
*Leptomeson dombrowskii pubipenne*		✔					
*Leptomeson svircevi knirschi*		✔					
*Leptomeson svircevi svircevi*		✔					
*Oryotus schmidtii schmidtii*							✔
*Oryotus schmidtii subdentatus*			✔				✔
*Parapropus ganglbaueri ganglbaueri*		✔					
*Parapropus ganglbaueri humeralis*		✔					
*Parapropus ganglbaueri obenbergeri*		✔					
*Parapropus ganglbaueri weiratheri*		✔					
*Parapropus ganglbaueri zepcensis*		✔					
*Parapropus sericeus augustae*			✔				
*Parapropus sericeus intermedius*			✔				
*Parapropus sericeus minutus*			✔				
*Parapropus sericeus muelleri*		✔					
*Parapropus sericeus sericeus*							✔
*Parapropus sericeus simplicipes*		✔					
*Parapropus sericeus sinuaticollis*		✔	✔				
*Parapropus sericeus stilleri*			✔				
*Parapropus sericeus taxi*			✔				
*Pholeuonella erberii epidaurica*			✔				
*Pholeuonella erberii erberii*		✔	✔		✔		
*Pholeuonopsis grabowskii grabowskii*		✔					
*Pholeuonopsis grabowskii lupoglavensis*		✔					
*Pholeuonopsis grabowskii ottonis*		✔					
*Pholeuonopsis grabowskii setniki*		✔					
*Pholeuonopsis setipennis fuchsi*		✔					
*Pholeuonopsis setipennis setipennis*		✔					
*Pretneria metkae metkae*							✔
*Pretneria metkae mirae*							✔
*Pretneria saulii montismusii*				✔			
*Pretneria saulii saulii*				✔			✔
*Proleonhardella matzenaueri matzenaueri*		✔					
*Proleonhardella matzenaueri ottonis*		✔					
*Redensekia likana kosiniensis*			✔				
*Redensekia likana likana*			✔				
*Spelaeobates coriniensis nonveilleri*			✔				
*Spelaeobates pharensis langhofferi*			✔				
*Spelaeobates pharensis pharensis*			✔				
*Speonesiotes dorotkanus dorotkanus*		✔	✔		✔		
*Speonesiotes dorotkanus noesskei*					✔		
*Speonesiotes koritoensis brevicornis*		✔					
*Speonesiotes koritoensis koritoensis*		✔					
*Speonesiotes koritoensis planaensis*		✔					
*Speonesiotes narentinus latitarsis*		✔	✔				
*Speonesiotes narentinus narentinus*		✔	✔				
*Speonesiotes narentinus simplicipes*		✔			✔		
*Speonesiotes remyi divinensis*		✔					
*Speonesiotes remyi remyi*		✔					
*Speonesiotes remyi winkleri*		✔					
*Tartariella durmitorensis durmitorensis*					✔		
*Tartariella durmitorensis zephyrensis*					✔		
